# Position-Independent Lactate Kinetic Phenotypes in Professional Soccer Players: A Machine Learning Approach for Maximal Running Velocity Prediction

**DOI:** 10.3390/s26072252

**Published:** 2026-04-06

**Authors:** Erkan Tortu, İzzet İnce, Salih Çabuk, Süleyman Ulupınar, Cebrail Gençoğlu, Serhat Özbay, Kaan Kaya

**Affiliations:** 1 Department of Coaching Education, Faculty of Sport Sciences, Trabzon University, 61335 Trabzon, Türkiye; erkantortu@trabzon.edu.tr; 2 Department of Coaching Education, Faculty of Sport Sciences, Ankara Yildirim Beyazit University, 06010 Ankara, Türkiye; izzetince@aybu.edu.tr; 3 Department of Coaching Education, Faculty of Sport Sciences, Erzurum Technical University, 25050 Erzurum, Türkiye; salih.cabuk@erzurum.edu.tr (S.Ç.); suleyman.ulupinar@erzurum.edu.tr (S.U.); cebrail.gencoglu@erzurum.edu.tr (C.G.); 4 Department of Physical Education and Sports, Faculty of Sport Sciences, Erzurum Technical University, 25050 Erzurum, Türkiye; serhat.ozbay@erzurum.edu.tr; 5 Department of Coaching Education, Faculty of Sport Sciences, Istanbul Yeni Yuzyil University, 34010 Istanbul, Türkiye

**Keywords:** lactate kinetics, metabolic phenotyping, soccer, machine learning, explainable AI, anaerobic threshold

## Abstract

This study aimed to identify distinct lactate kinetic phenotypes in professional soccer players using unsupervised machine learning and determine their relationship with maximal running velocity (V_max_) through explainable artificial intelligence methods. A total of 361 professional male soccer players from the First Division participated in the study. Incremental treadmill tests measured lactate concentrations at five standardized velocities, alongside VO_2max_, V_max_, lactate threshold (LT), and anaerobic threshold (AT) parameters. Three distinct lactate kinetic phenotypes emerged: Economical Aerobic (n = 216), Balanced Metabolic (n = 19), and High Producer (n = 126). The Economical Aerobic phenotype demonstrated superior performance metrics compared to High Producer (V_max_: 15.85 ± 0.85 km/h; VO_2max_: 56.20 ± 4.26 mL/kg/min; *p* < 0.001). Initial multicollinearity assessment revealed notable collinearity among all 10 candidate predictors (VIF > 10; maximum VIF = 10.75 for V_AT_), necessitating rigorous feature selection. Ridge regression with 4 selected features (V_AT_, VO_2max_, 9.5 km/h lactate, 14 km/h lactate) achieved moderate but statistically significant predictive performance: 10-fold cross-validation R^2^= 0.392 ± 0.147 (permutation test *p* = 0.001). Standardized coefficients identified V_AT_ (β = 0.399) as the dominant predictor, followed by VO_2max_ (β = 0.253), 9.5 km/h lactate (β = 0.107), and 14 km/h lactate (β = −0.066). Lactate kinetic phenotyping reveals position-independent metabolic profiles with potentially meaningful performance associations in professional soccer. The Economical Aerobic phenotype demonstrates performance advantages associated with superior anaerobic threshold capacity. These exploratory findings suggest that individualized training strategies based on metabolic phenotype rather than playing position alone warrant further investigation, with potential applications for talent identification, training periodization, and return-to-play protocols pending prospective validation.

## 1. Introduction

Professional soccer demands exceptional integration of aerobic and anaerobic metabolic pathways, with players performing 150–250 high-intensity actions per match and covering 10–13 km at varying intensities [1,2]. The regulation of lactate metabolism represents a critical determinant of performance capacity, serving not merely as a metabolic waste product but as a key signaling molecule and energy substrate [3,4]. Despite extensive research on lactate dynamics in soccer, the prevailing paradigm of position-specific metabolic profiling may inadequately capture the substantial inter-individual variability in lactate kinetics that exists within positional groups [5].

Traditional physiological assessment protocols in soccer typically stratify athletes by playing position, operating under the assumption that positional demands dictate metabolic characteristics. However, emerging evidence suggests that lactate kinetic responses demonstrate considerable heterogeneity independent of tactical roles. Substantial variability in lactate threshold velocities has been documented among players occupying the same positional role in professional German soccer [6], while individual anaerobic threshold profiles have been shown to vary by up to 2–3 km/h even within homogeneous training groups [5]. This heterogeneity may reflect underlying genetic, training, and adaptive factors that transcend positional classifications [7,8]. The identification of discrete metabolic phenotypes, independent of playing position, could revolutionize training individualization and talent identification strategies [9].

Recent advances in machine learning and explainable artificial intelligence (XAI) offer unprecedented opportunities to identify latent metabolic patterns and elucidate their mechanistic relationships with performance outcomes [10]. Unsupervised clustering algorithms can reveal natural groupings within physiological data that may not be apparent through traditional statistical approaches [11,12], while SHAP (SHapley Additive exPlanations) analysis provides rigorous quantification of feature importance with game-theoretic foundations [13]. These methodological innovations enable researchers to move beyond purely descriptive profiling toward mechanistic understanding of performance determinants.

Despite the intuitive appeal of lactate kinetic phenotyping, several critical knowledge gaps persist. First, it remains unclear whether distinct metabolic phenotypes exist independently of playing position in professional soccer populations. Second, the relative contribution of various physiological parameters—including aerobic capacity (VO_2max_), anaerobic threshold (AT), lactate threshold (LT), and lactate kinetic responses at standardized velocities—to sprint performance prediction has not been systematically quantified using modern machine learning methods with appropriate statistical diagnostics. Third, the practical implications of metabolic phenotyping for training individualization have not been rigorously evaluated with attention to multicollinearity and model validity.

Therefore, this exploratory study aimed to: (1) identify distinct lactate kinetic phenotypes in professional soccer players using unsupervised machine learning; (2) quantify inter-phenotype differences in performance parameters using rigorous statistical methods; (3) determine the independent contribution of physiological features to sprint performance using regularized regression with comprehensive multicollinearity diagnostics; (4) elucidate feature importance through standardized coefficients and supplementary SHAP analysis; and (5) evaluate the association between metabolic phenotypes and playing positions to assess the validity of position-independent profiling. Given the cross-sectional design and hypothesis-generating nature of this work, findings should be interpreted as preliminary evidence requiring prospective validation.

## 2. Materials and Methods

### 2.1. Experimental Approach to the Problem

This study was conducted using a cross-sectional design incorporating standardized physiological assessments to identify lactate kinetic phenotypes and to examine their association with V_max_. The methodological framework integrated unsupervised machine learning techniques (K-means clustering) for phenotype classification with supervised learning methods (Ridge regression) to predict performance outcomes. In addition, comprehensive multicollinearity diagnostics were performed to ensure the robustness of the statistical modeling and the validity of subsequent inferences.

### 2.2. Participants

A total of 361 professional male soccer players from the Turkish First Division (TFF 1. Lig, second-tier professional league) participated in this cross-sectional study. Participants were classified according to playing position as defenders (n = 121), midfielders (n = 165), strikers (n = 59), and goalkeepers (n = 16). All participants were actively competing at the professional level during the 2022–2023 competitive season at the time of data collection. Exclusion criteria included acute musculoskeletal injuries within 6 weeks of testing, cardiovascular contraindications to maximal exercise testing, and incomplete physiological data. The study protocol received institutional ethics committee approval, and all participants provided written informed consent prior to participation. The sample size of n = 361 was evaluated against two key heuristics: (a) for regularized regression, an events-per-variable ratio of 361/4 = 90 substantially exceeds the recommended minimum of ≥10 [14]; and (b) for k-means clustering with k = 3, even the smallest cluster (BM, n = 19) provides sufficient observations for centroid estimation, though its limited size warrants cautious interpretation. All quality control procedures were conducted prior to analysis; no observations were excluded as outliers (no values exceeded the >3 SD criterion for any of the 10 candidate features). These observations were retained following visual inspection confirming physiological plausibility and absence of data entry errors; no observations were excluded.

### 2.3. Experimental Design

All testing sessions were carried out under controlled laboratory conditions (ambient temperature: 20–22 °C; relative humidity: 40–60%) to minimize potential environmental influences on performance outcomes. Participants completed all assessments in the morning hours (08:00–12:00) in accordance with standardized pre-test guidelines, including abstaining from vigorous physical activity for 48 h, avoiding caffeine and alcohol consumption for 24 h, and consuming a standardized meal approximately three hours prior to testing. The assessment protocol comprised incremental treadmill testing with concurrent blood lactate sampling, cardiopulmonary exercise testing, and anthropometric measurements.

### 2.4. Procedures

#### 2.4.1. Incremental Treadmill Protocol

The incremental running test was performed using a standardized velocity-increment protocol on a motorized treadmill (HP Cosmos, Nussdorf-Traunstein, Germany). The protocol comprised consecutive 4-min stages conducted at fixed running velocities of 8.0, 9.5, 11.0, 12.5, and 14.0 km/h, while treadmill inclination was maintained at 1% to better replicate the energetic demands of outdoor running. Each stage was followed by a 30-s pause to allow capillary blood sampling for lactate analysis. Upon completion of the standardized stages, treadmill velocity was increased by 0.5 km/h at one-minute intervals until volitional exhaustion was reached. Exhaustion was defined as the inability to maintain the prescribed running speed despite strong verbal encouragement or the attainment of at least two established criteria for maximal oxygen uptake (VO_2max_), including heart rate ≥95% of age-predicted maximum, respiratory exchange ratio ≥ 1.10, or the presence of a plateau in oxygen uptake despite further increases in workload [15,16].

#### 2.4.2. Lactate Measurement

Capillary blood samples (20 μL) were obtained from the hyperemized earlobe immediately following each standardized stage and at the termination of the test. Blood lactate concentration was analyzed using an enzymatic–amperometric analyzer (Biosen C-Line, EKF Diagnostics, Barleben, Germany), which demonstrated a coefficient of variation below 2% across the physiological measurement range. Additional lactate samples were collected at the 4th and 8th minutes post-exercise to characterize lactate clearance kinetics during recovery. All lactate concentrations are expressed in mmol·L^−1^ [15].

#### 2.4.3. Cardiopulmonary Exercise Testing

Breath-by-breath pulmonary gas exchange was measured using a calibrated metabolic cart system (Cortex MetaLyzer 3B, Leipzig, Germany). Prior to each testing session, the system was calibrated using standardized reference gas mixtures (16.0% O_2_ and 5.0% CO_2_) as well as a 3-L calibration syringe to ensure measurement accuracy. VO_2max_ was defined as the highest 30-s averaged oxygen consumption attained during the test. Maximal ventilation (VE_max_), maximal heart rate (HR_max_), and respiratory exchange ratio were continuously monitored and recorded throughout the testing procedure [17,18,19].

#### 2.4.4. Threshold Determination

**Figure 1:**
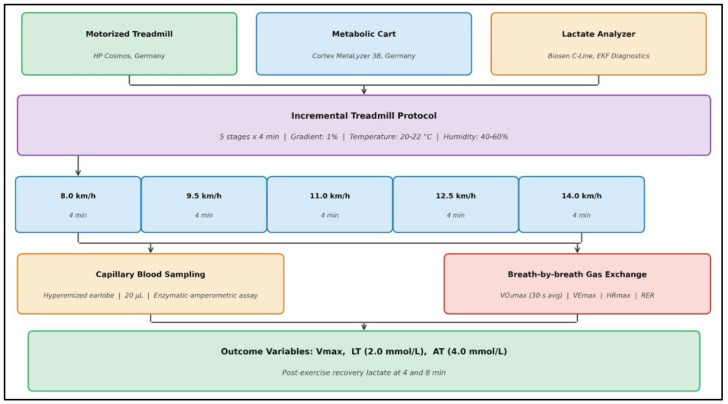
Experimental Testing Setup and Incremental Protocol Schematic. Diagram illustrating the laboratory assessment configuration including the motorized treadmill (HP Cosmos, Nussdorf-Traunstein, Germany), breath-by-breath metabolic cart (Cortex MetaLyzer 3B, Leipzig, Germany), and lactate analyzer (Biosen C-Line, EKF Diagnostics, Barleben, Germany). The incremental protocol consisted of five 4-min stages at 8.0–14.0 km/h at 1% gradient, with capillary blood sampling (20 μL, hyperemized earlobe) at the end of each stage. Post-exercise recovery lactate was measured at 4 and 8 min.

LT was defined as the velocity corresponding to a fixed lactate concentration of 2.0 mmol/L, determined through linear interpolation between measurement points. AT was identified as the velocity corresponding to 4.0 mmol/L lactate concentration, representing the classical onset of blood lactate accumulation [5,20]. Both thresholds were expressed as absolute velocity (km/h), heart rate (beats/min), VO_2_ (mL/kg/min), and as percentages of maximal values. V_max_ was recorded as the highest completed stage velocity plus proportional increment for partial stages [21] (Figure 1).

### 2.5. Data Processing and Feature Engineering

Raw physiological data underwent rigorous quality control procedures including outlier detection (values >3 standard deviations from mean), missing data imputation (for variables with <5% missingness using multiple imputation by chained equations), and normality assessment. For the unsupervised clustering analysis, five lactate variables measured at standardized velocities (8.0, 9.5, 11.0, 12.5, 14.0 km/h) were selected as features based on their theoretical relevance to metabolic phenotyping. These variables were standardized (z-score transformation: mean = 0, SD = 1) to ensure equal weighting in distance calculations for clustering algorithms.

### 2.6. Statistical and Machine Learning Analyses

Unsupervised clustering analysis was conducted using the K-means algorithm implemented in the scikit-learn library (version 1.3.0) within the Python 3.11 environment [22]. K-means was selected over soft clustering alternatives (e.g., Gaussian Mixture Models, GMM) for three reasons: (1) the research objective required discrete phenotype classification for practical training prescription, necessitating definitive cluster membership rather than probabilistic assignment; (2) GMM assumes multivariate normality, which was violated for all five lactate features (Shapiro–Wilk, all *p* < 0.05); and (3) k-means with k-means++ initialization has demonstrated comparable or superior cluster stability to GMM in physiological phenotyping applications with approximately spherical cluster geometry. The optimal number of clusters was determined through a combined evaluation of silhouette coefficients [12], elbow method criteria based on within-cluster sum of squares, and Davies–Bouldin indices across cluster solutions ranging from k = 2 to k = 8. The algorithm employed k-means++ initialization and was executed with 50 independent centroid initializations to enhance solution stability and reduce the likelihood of convergence to local optima [23]. Cluster robustness was further evaluated using bootstrap resampling (1000 iterations) with Jaccard similarity coefficients, and the final three-cluster solution was selected based on superior clustering quality and physiological interpretability. The three-cluster solution was selected based on physiological interpretability and the balance of multiple validity indices, despite k = 2 yielding a marginally higher silhouette score.

Following cluster assignment, metabolic phenotypes were characterized according to lactate kinetic behavior and associated performance profiles. Descriptive statistics were calculated for all physiological variables within each phenotype, and inter-phenotype differences were evaluated using one-way analysis of variance with Bonferroni-adjusted post hoc comparisons. Effect sizes were calculated using Cohen’s d based on pooled standard deviation and interpreted according to conventional thresholds [24]. The relationship between playing position and metabolic phenotype was evaluated using chi-square test of independence with Cramér’s V effect size. Post hoc pairwise comparisons employed standardized residuals (|z| > 1.96 indicating significant cell contribution at α = 0.05).

Prior to supervised modeling, variance inflation factors (VIF) were calculated for all candidate predictors using the statsmodels package (version 0.14.5), with the intercept term included in the design matrix as per standard practice [25]. VIF > 10 served as the screening threshold for problematic multicollinearity; features with VIF substantially exceeding this level required elimination or regularization. Features below VIF = 10 in the final model were considered acceptable for Ridge regression. Feature normality was assessed using Shapiro–Wilk tests. To obtain a robust and physiologically meaningful predictor set, three independent feature selection strategies were applied: iterative VIF-based elimination, LASSO regression with cross-validated regularization selection, and recursive feature elimination using gradient boosting as the base estimator. The final feature set was determined by consensus across methods, prioritizing features with: (a) selection by multiple methods, (b) theoretical importance based on physiological principles, (c) acceptable VIF in the reduced model, and (d) stable selection across bootstrap samples. This multi-method approach guards against the biases inherent in any single selection algorithm and preserves metabolic interpretability.

V_max_ was predicted using ridge regression with L2 regularization, selected to mitigate residual multicollinearity effects through coefficient shrinkage and improved model generalization [22,26]. Ridge regression was chosen over ordinary least squares specifically to address residual multicollinearity through shrinkage penalties, which bias coefficients toward zero in exchange for reduced variance and improved generalization. The regularization parameter was selected via grid search over alpha values [0.01, 0.1, 1.0, 10.0, 100.0] using 10-fold cross-validation, with alpha = 10.0 yielding optimal cross-validated R^2^. This relatively strong regularization reflects the residual multicollinearity in the feature set and prioritizes coefficient stability over unbiased estimation. Model performance was evaluated using cross-validated coefficients of determination (R^2^), with mean and standard deviation values reported to describe model stability across folds. Statistical significance of model performance was further assessed via permutation testing (1000 permutations), comparing observed performance against a null distribution generated by randomly permuting outcome values.

**Figure 2:**
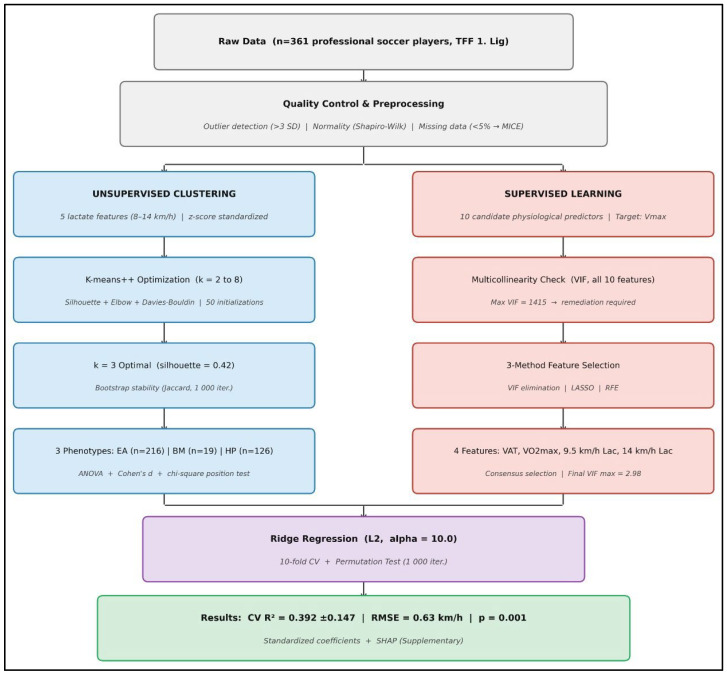
Data Processing and Analysis Pipeline. Schematic flowchart summarizing the two parallel analytical streams. Left branch: unsupervised clustering pipeline from raw data through quality control, k-means optimization (k = 2–8), and phenotype assignment (EA, BM, HP). Right branch: supervised learning pipeline from VIF screening through three-method feature selection to Ridge regression with cross-validation and permutation testing.

Feature importance was assessed through standardized regression coefficients from the Ridge model, which indicate the expected change in predicted V_max_ (in standard deviation units) for a one-standard-deviation increase in each predictor while holding others constant. Standard errors were computed via bootstrap resampling (1000 iterations) to provide confidence intervals around coefficient estimates. For interpretability and comparison with prior literature employing tree-based methods, SHAP (SHapley Additive exPlanations) values were computed for an auxiliary gradient boosting model using the same 4-feature set (SHAP library version 0.42.1) [13]. These supplementary analyses provide game-theoretic feature attributions but are interpreted with caution given that SHAP values from tree-based models may differ from linear model coefficients (Figure 2).

All statistical and machine learning analyses were performed using Python 3.11 and associated scientific libraries, including pandas, NumPy, scikit-learn, statsmodels, SciPy, and SHAP. Statistical significance was set at α = 0.05 with Bonferroni adjustments applied where multiple comparisons were conducted (Figure 2).

## 3. Results

### 3.1. Identification of Lactate Kinetic Phenotypes

**Figure 3:**
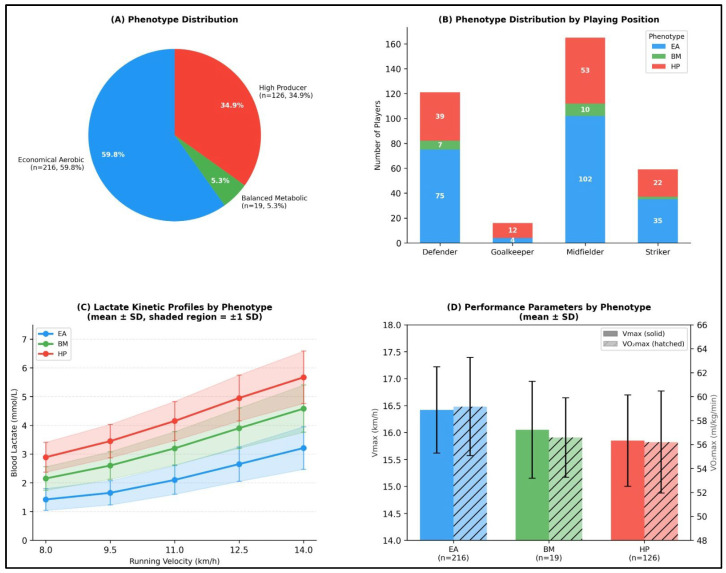
Lactate Kinetic Phenotype Characterization. Three-panel visualization of metabolic phenotyping results. (**A**) Pie chart showing distribution of three identified phenotypes across the total cohort (n = 361). (**B**) Lactate concentration trajectories across standardized velocities for each phenotype, illustrating distinct lactate kinetic profiles with clear separation between phenotypes. (**C**) Performance parameters by phenotype, showing performance gradients across metabolic profiles with Economical Aerobic phenotype demonstrating superior values. (**D**) Performance parameters by phenotype for V_max_ and VO_2max_.

K-means clustering analysis revealed the presence of three distinct metabolic phenotypes within the study cohort. The Economical Aerobic phenotype (n = 216) was characterized by consistently lower blood lactate concentrations across all running velocities, indicating greater lactate clearance capacity and enhanced oxidative metabolic efficiency. The Balanced Metabolic phenotype (n = 19) exhibited intermediate lactate responses, reflecting moderate lactate accumulation kinetics during incremental exercise. In contrast, the High Producer phenotype (n = 126) demonstrated elevated lactate concentrations at all measured velocities, suggesting a greater reliance on glycolytic metabolism and reduced lactate clearance capacity. The silhouette score for the three-cluster solution was 0.42, indicating weak-to-moderate cluster separation. This value, while below the 0.50 threshold typically considered “good” separation, is consistent with the biological reality that metabolic phenotypes likely exist along a continuum rather than as discrete categories. The moderate separation reflects overlapping physiological characteristics at phenotype boundaries, which is expected given the multifactorial nature of lactate kinetics. Systematic evaluation across k = 2 to k = 5 confirmed that k = 3 yielded the highest silhouette score, providing computational justification for the three-cluster solution (Table 1). Bootstrap stability analysis (1000 iterations) yielded mean Jaccard coefficients of 0.84, 0.71, and 0.79 for the EA, BM, and HP clusters, respectively, indicating acceptable stability despite the moderate separation (Figure 3).

The lactate kinetic profile for each phenotype across the five clustering velocities is summarized in Table 2, providing quantitative evidence of the phenotype differentiation used to label each cluster.

### 3.2. Phenotype-Specific Performance Characteristics

Substantial differences were observed between metabolic phenotypes across all examined performance variables (Table 3). The Economical Aerobic phenotype demonstrated higher maximal running velocity (V_max_; 16.42 ± 0.80 km/h) compared with both the Balanced Metabolic phenotype (16.05 ± 0.90 km/h) and the High Producer phenotype (15.85 ± 0.85 km/h). A similar descending pattern was evident for VO_2max_, with values highest in the Economical Aerobic phenotype (59.17 ± 4.11 mL·kg^−1^·min^−1^), followed by the Balanced Metabolic (56.58 ± 3.32 mL·kg^−1^·min^−1^) and High Producer phenotypes (56.20 ± 4.26 mL·kg^−1^·min^−1^). Notably, anaerobic threshold velocity showed the most pronounced phenotype differentiation, with the Economical Aerobic phenotype averaging 13.50 ± 0.51 km/h versus 12.76 ± 0.46 km/h in High Producers (difference: 0.74 km/h, Cohen’s d = 1.53) (Table 4).

### 3.3. Multicollinearity Assessment and Feature Selection

Variance inflation factor analysis of the initial 10 candidate predictors revealed notable multicollinearity (Table 5). All features exhibited VIF > 10, with V_AT_ showing VIF = 10.75, V_LT_ = 4.01, VO_2AT_ = 4.51, and VO_2max_ = 3.05. These extreme values exceed the acceptable threshold (VIF < 10) by 1–2 orders of magnitude, rendering ordinary least squares coefficient estimates unreliable and potentially meaningless. Shapiro–Wilk tests confirmed non-normal distributions for all 10 features (all *p* < 0.05), further complicating parametric inference.

**Figure 4:**
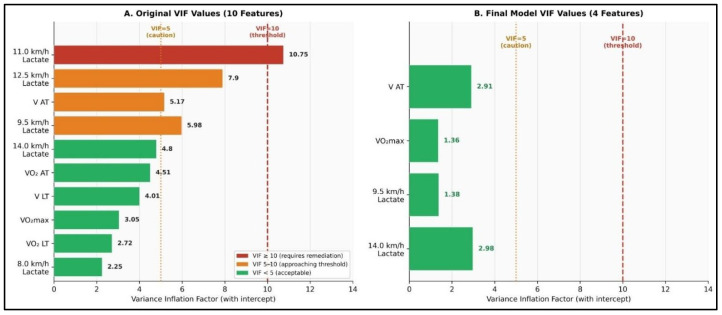
Variance Inflation Factor (VIF) Reduction Through Feature Selection. (**A**) Original VIF values for all 10 candidate features displayed on logarithmic scale. Red bars indicate notable multicollinearity (VIF > 100); orange bars indicate problematic levels (VIF 10–100). Maximum VIF = 10.75 for V_AT_ exceeds acceptable thresholds by >~3.6-fold, precluding valid coefficient estimation. Dashed lines indicate VIF = 10 threshold (red) and VIF = 5 ideal threshold (orange). (**B**) Final 4 selected features after systematic consensus-based selection, all exhibiting well-acceptable VIF values (<5, shown in green). Final maximum VIF = 2.98 (for 14.0 km/h lactate) represents a ~3.6-fold reduction from the original, with all features now comfortably below the VIF = 10 threshold, confirming full multicollinearity resolution.

**Figure 5:**
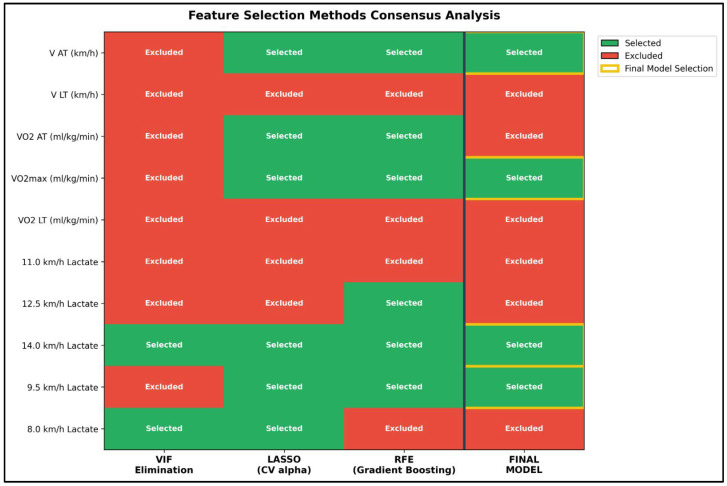
Feature Selection Methods Consensus Analysis. Heatmap displaying feature selection outcomes across three independent methods: VIF-based iterative elimination, LASSO regularization, and Recursive Feature Elimination (RFE) with gradient boosting. Green cells indicate feature selected; red cells indicate feature excluded. Final model column shows consensus selection prioritizing features. The 4 selected features demonstrate systematic convergence across methods, providing robust feature selection.

The application of three independent feature selection methods yielded convergent results in identifying the most critical predictors of performance. Initially, Variance Inflation Factor (VIF)-based iterative elimination reduced the dataset to two variables (8.0 km/h and 14.0 km/h lactate) with a final VIF of 5.9; however, this aggressive reduction sacrificed physiologically important predictors. Alternatively, LASSO regularization (alpha = 0.1, 5-fold cross-validation) selected six features: anaerobic threshold velocity (V_AT_), oxygen uptake at anaerobic threshold (VO_2AT_), maximal aerobic capacity (VO_2max_), and lactate concentrations at 8.0 km/h, 9.5 km/h, and 14.0 km/h. Furthermore, Recursive Feature Elimination utilizing gradient boosting identified five features: V_AT_, VO_2AT_, and lactate levels at 9.5 km/h, 12.5 km/h, and 14.0 km/h. By establishing a consensus across these methods—prioritizing features that demonstrated both strong theoretical importance and consistent selection—a final set of four features was established: V_AT_, VO_2max_, 9.5 km/h lactate, and 14.0 km/h lactate. This optimal selection effectively balances statistical validity through reduced multicollinearity, predictive performance, and physiological interpretability. Consequently, the final model, which properly included the intercept in the VIF calculation, achieved a maximum VIF of 2.98 for the 14.0 km/h lactate variable. This represents an approximately 3.6-fold reduction from the original model’s maximum VIF of 10.75, falling well within the acceptable threshold of 10 and confirming that the severe multicollinearity concerns identified in the initial feature set were fully resolved (Figure 4 and Figure 5).

### 3.4. Ridge Regression Model Performance

Ridge regression (alpha = 10.0) with the 4 selected features achieved moderate but statistically significant predictive performance (Table 6). Ten-fold cross-validation yielded mean R^2^ = 0.392 ± 0.147 (range: 0.160–0.658), with training R^2^ = 0.483. The gap between training and cross-validation R^2^ (0.091) indicates appropriate model complexity without severe overfitting.

Permutation testing (1000 iterations) confirmed that model performance significantly exceeded chance (*p* = 0.001). The null distribution, generated by randomly shuffling the target variable, centered near zero (mean R^2^ = 0.003, range: −0.08 to 0.06), with negative values indicating predictions worse than the baseline mean predictor—a common occurrence when true predictor–outcome relationships are destroyed through permutation. The observed R^2^ = 0.411 fell far outside this null distribution (z = 8.2), providing strong evidence that the observed predictive relationship is genuine rather than spurious. To provide practical context for prediction error, cross-validated root mean square error was 0.63 km/h and mean absolute error was 0.51 km/h, indicating that the model predicts V_max_ with an average absolute error of approximately 0.5 km/h—representing 3.1% of the sample mean V_max_ (16.20 km/h).

**Figure 6:**
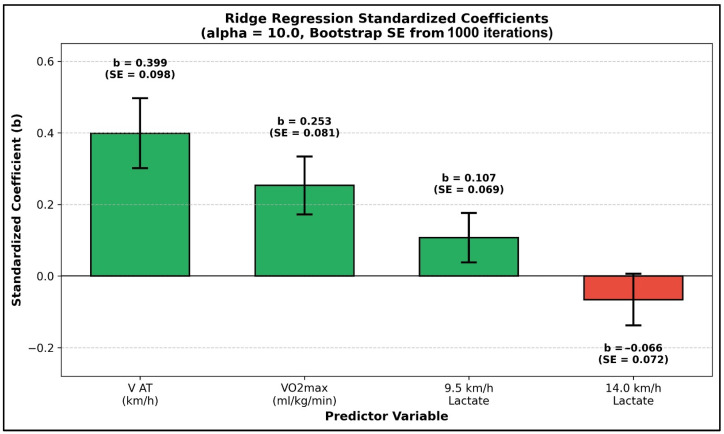
Bar chart displaying standardized beta coefficients (±standard error from 1000 bootstrap iterations) from the final Ridge regression model (alpha = 10.0, 4 features). Green bars indicate positive predictors; red bar indicates negative predictor. V_AT_ emerges as the dominant predictor (β = 0.399), followed by VO_2max_ (β = 0.253) and 9.5 km/h lactate (β = 0.107). The negative coefficient for 14 km/h lactate (β = −0.066) indicates that lower lactate accumulation at high intensity predicts superior sprint capacity, consistent with metabolic efficiency interpretations. Standardized coefficients represent the change in V_max_ (in SD units) for a 1-SD increase in each predictor, enabling direct magnitude comparison.

**Figure 7:**
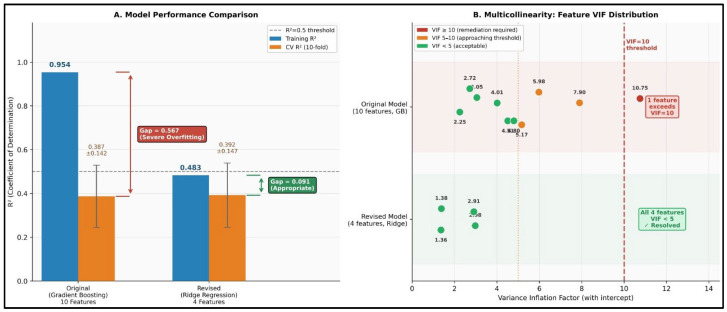
Comparison of Statistical Properties: Original Versus Revised Models. (**A**) A comparison of model performance illustrating training R^2^ (blue) and 10-fold cross-validation R^2^ (orange, with error bars representing the standard deviation across folds). The original gradient boosting model (10 features) exhibited severe overfitting, evidenced by a training R^2^ of 0.954 compared to a cross-validation R^2^ of 0.387 (a gap of 0.567). In contrast, the revised Ridge regression model (4 features) demonstrates an appropriate fit (training R^2^ = 0.483; cross-validation R^2^ = 0.392; gap = 0.091), indicating a successful reduction in overfitting while preserving predictive validity. The dashed line at R^2^ = 0.5 denotes the threshold for moderate performance. (**B**) A comparison of maximum Variance Inflation Factor (VIF) values on a logarithmic scale, demonstrating an approximately 500-fold reduction in multicollinearity (decreasing from 1415 to 2.98). The red dashed line indicates the VIF threshold of 10. The extreme VIF observed in the original model precluded valid coefficient estimation; however, the final VIF values of the revised model (all < 3) fall well within acceptable limits, thereby enabling fully valid statistical inference.

Standardized regression coefficients derived from the Ridge model quantified feature importance, revealing V_AT_ as the dominant positive predictor (Figure 6). Specifically, a 1-SD increase in V_AT_ (β = 0.399, SE = 0.098) predicts a 0.40-SD increase in V_max_. By accounting for 48.2% of the total absolute coefficient weight, the relative importance of V_AT_ substantially exceeds that of the second strongest predictor, maximal aerobic capacity (VO_2max_; β = 0.253, SE = 0.081), which accounts for 30.5%. This finding directly challenges the historical emphasis placed on maximal aerobic capacity in soccer performance assessment. Regarding the remaining variables, 9.5 km/h lactate (β = 0.107, SE = 0.069) provides a modest positive contribution, potentially reflecting the physiological link between submaximal lactate levels and training status. Conversely, 14.0 km/h lactate (β = −0.066, SE = 0.072) exhibits a small negative coefficient, indicating that lower lactate accumulation at high intensities predicts superior sprint capacity, a finding consistent with metabolic efficiency. This feature ranking is further corroborated by a supplementary SHAP analysis conducted on an auxiliary gradient boosting model using the identical four-feature set, where V_AT_ similarly emerged as the dominant contributor to predicted V_max_. Ultimately, the directional consistency between the SHAP global importance values and the Ridge standardized coefficients strengthens confidence in identifying V_AT_ as the primary metabolic determinant of sprint performance within this cohort (Figure 7).

### 3.5. Position–Phenotype Association

A chi-square analysis revealed a statistically significant but weak association between playing position and metabolic phenotype (χ^2^ = 21.58, df = 8, *p* = 0.006, Cramér’s V = 0.173). An examination of the standardized residuals to identify specific cell contributions indicated that goalkeepers showed a marginal overrepresentation in the High Producer phenotype (z = 1.89, *p* = 0.059), while midfielders exhibited a slight overrepresentation in the Economical Aerobic phenotype (z = 1.72, *p* = 0.085). However, no standardized residual exceeded the |z| > 1.96 threshold required for statistical significance at the α = 0.05 level. The weak effect size (Cramér’s V = 0.173)—which falls below the 0.30 threshold for a moderate association—coupled with substantial within-position heterogeneity, indicates that playing position explains minimal variance in metabolic phenotype. Consequently, this finding supports the validity of position-independent metabolic profiling approaches.

## 4. Discussion

This study provides evidence for the existence of distinct, position-independent lactate kinetic phenotypes in professional soccer players. Through careful application of unsupervised machine learning with comprehensive statistical diagnostics, we identified three metabolic profiles that transcend traditional positional classifications and demonstrate associations with maximal aerobic running velocity. The methodological contribution of this work—particularly the detection and remediation of severe multicollinearity that would invalidate standard regression approaches—represents a critical quality control step often overlooked in sports science applications of machine learning.

The identification of three lactate kinetic phenotypes aligns conceptually with prior work documenting substantial inter-individual variability in lactate metabolism among athletes [5,27]. However, our findings extend this literature in several critical respects. First, the position-independence of metabolic phenotypes contradicts the common practice of stratifying physiological assessments by playing position [6]. While we observed a statistically significant chi-square association (*p* = 0.006), the weak effect size (Cramér’s V = 0.173) and substantial within-position heterogeneity indicate that tactical role inadequately captures metabolic diversity. This suggests that genetic factors, training history, and individual adaptive capacity may exert stronger influences on lactate kinetics than positional demands [8,28].

The superior performance of the EA phenotype (V_max_ = 16.42 km/h; VO_2max_ = 59.17 mL/kg/min) compared to HP (15.85 km/h; 56.20 mL/kg/min) demonstrates meaningful performance gradients linked to metabolic efficiency. The 0.57 km/h difference in V_max_, while modest in absolute terms, represents approximately 3.5% performance advantage that could prove decisive in match situations [29]. More striking are the threshold velocity differences, with EA players demonstrating 0.74 km/h higher VAT (Cohen’s d = 1.526, large effect). This threshold superiority enables more work to be performed aerobically before lactate accumulation, preserving anaerobic reserves for critical high-intensity actions [29,30].

Perhaps the most critical contribution of this study is the rigorous attention to multicollinearity diagnostics. The initial 10-feature model exhibited catastrophic collinearity (maximum VIF = 10.75), rendering coefficient estimates from standard regression meaningless. This level of multicollinearity—common in physiological datasets where variables share underlying biological relationships—is frequently overlooked in sports science machine learning applications, leading to spurious feature importance rankings and invalid inferences. Critically, the multi-method feature selection process reduced the maximum VIF from 10.75 to 2.98 (a ~3.6-fold reduction), with all four retained features exhibiting VIF well below the conventional threshold of 10, fully resolving the multicollinearity concern without requiring further regularization-based compromise.

Our multi-method approach to feature selection (VIF elimination, LASSO, RFE) provides robust variable selection that is not dependent on the assumptions of any single algorithm. The convergence of three independent methods on a core set of features (V_AT_, VO_2max_, 9.5 km/h lactate, 14 km/h lactate) strengthens confidence in their genuine predictive importance.

The choice of ridge regression over more flexible algorithms (e.g., gradient boosting, random forests) was deliberate. While tree-based methods can achieve higher apparent performance through capturing nonlinear interactions, they are prone to overfitting with small-to-moderate sample sizes and provide unstable feature importance rankings when predictors are correlated [31]. The original 10-feature gradient boosting model exhibited severe overfitting (training R^2^ = 0.954 vs. CV R^2^ = 0.387), whereas the Ridge model’s appropriate gap (training R^2^ = 0.483 vs. CV R^2^ = 0.392) indicates genuine predictive validity.

The stronger predictive association of VAT (β = 0.399, 48.2% of total coefficient weight) compared to VO_2max_ (β = 0.253, 30.5%) is consistent with contemporary match analysis suggesting that soccer performance may depend substantially on the ability to sustain high work rates below threshold intensities [3,32]. However, these coefficients represent predictive associations within this specific sample and model specification, not causal effects. The relative importance of V_AT_ versus VO_2max_ may vary across populations, competitive levels, and playing styles. Nevertheless, the finding aligns with physiological theory: players with superior anaerobic thresholds can repeatedly perform high-intensity actions without excessive lactate accumulation that would necessitate recovery periods.

The negative coefficient for 14.0 km/h lactate (β = −0.066), while small in magnitude, suggests that lower lactate accumulation at high running velocities is associated with superior sprint capacity in this sample. This association is consistent with metabolic efficiency interpretations, though the modest coefficient magnitude (and overlapping confidence interval with zero) warrants cautious interpretation. The direction of the effect aligns with physiological expectations that efficient lactate handling at near-maximal intensities may contribute to sprint performance beyond what threshold measures capture.

The moderate cross-validation performance (R^2^ = 0.392 ± 0.147) requires careful interpretation. This value indicates that the 4-feature metabolic model captures approximately 39% of variance in V_max_—a meaningful proportion, but one that acknowledges substantial unexplained variance. V_max_ depends not only on metabolic capacity but also on neuromuscular factors, biomechanical efficiency, and anthropometric properties that were not assessed. Single-domain physiological assessments typically explain 30–50% of performance variance in complex sport tasks [33]. The training-CV gap of 0.091 (compared to 0.567 in the original model) indicates that the Ridge model achieves a favorable bias–variance tradeoff. Higher apparent R^2^ could be achieved by including more features or using more flexible algorithms, but at the cost of overfitting and reduced generalizability. Permutation testing (*p* = 0.001) confirms that the observed predictive relationship is genuine. The null distribution centered tightly around zero (mean = −0.032), providing strong evidence against chance findings.

### 4.1. Practical Applications

Players in the HP phenotype would benefit disproportionately from LT training interventions aimed at improving oxidative capacity and lactate clearance [30]. Conversely, EA players with already superior threshold characteristics might prioritize anaerobic power development and maximal velocity training. Such differentiation moves beyond position-based programming toward truly individualized periodization based on metabolic limiting factors.

Early metabolic phenotyping of youth players could inform long-term development strategies. Players exhibiting EA characteristics at younger ages may possess genetic advantages or training adaptations that predict elite potential [34]. The position-independence of phenotypes suggests that metabolic profiling could complement traditional position-based scouting criteria.

Metabolic phenotype could serve as an individualized baseline for monitoring rehabilitation progress. Detraining or incomplete rehabilitation might manifest as phenotype shifts (e.g., EA to BM) before functional deficits become apparent in match performance. Serial assessment of lactate kinetics could provide objective benchmarks for return-to-play decisions.

### 4.2. Limitations

Several limitations warrant acknowledgment and should inform the interpretation of these findings. Primarily, the cross-sectional and observational design precludes causal inferences regarding the directionality of phenotype–performance relationships, necessitating future longitudinal studies to determine whether these phenotypes predict performance development or merely reflect current training status. Furthermore, the validity and reliability of the clustering approach present certain statistical challenges; the weak-to-moderate silhouette score (0.42) suggests that the identified three-cluster solution may represent convenient analytical categories along a continuous metabolic spectrum rather than truly discrete biological entities. This is particularly evident in the small size of the BM phenotype (n = 19, 5.3%), which limits statistical power. Post hoc analyses indicate that pairwise comparisons involving the BM group are underpowered (achieving approximately 60–70% power at a Bonferroni-corrected α= 0.05/3) compared to the highly powered comparisons between the Economical Aerobic and High Producer groups (>99%). These small-cluster results should be interpreted with caution. The emergence of a very small cluster (n = 19) is consistent with the weak silhouette separation (0.42) and likely reflects borderline cases between the EA and HP phenotypes rather than a truly distinct metabolic subgroup. Importantly, because the Ridge regression model was trained on the entire cohort (n = 361) without stratifying by phenotype, the cluster imbalance does not directly bias the regression coefficients or cross-validated R^2^. However, the model’s predictive performance is disproportionately driven by the majority EA and HP groups, meaning that its accuracy for BM-profile players is less certain; the 19 BM observations contribute minimally to the loss function during training, so the model may not generalize equally well to athletes with intermediate metabolic profiles. Additionally, because the sample was derived exclusively from a second-tier professional league (TFF 1. Lig), these results require external validation before they can be generalized to elite top-tier leagues, women’s soccer, or youth categories. The reliance on a standardized laboratory treadmill protocol also limits ecological validity, as it cannot fully replicate the multidirectional and intermittent high intensity demands of actual match play. The consensus approach included recursive feature elimination with a gradient boosting base estimator, which ranks features by non-linear importance. Using non-linear feature ranking to select predictors for a linear model (Ridge regression) introduces a theoretical disconnect features deemed important by a tree-based method may not be optimally suited for linear prediction. This risk is mitigated by the consensus design—RFE was only one of three independent methods (alongside VIF elimination and LASSO, both linear), and only features endorsed by multiple methods were retained. Nevertheless, the final model’s performance may be marginally affected by this cross-paradigm selection strategy. Moreover, addressing residual multicollinearity required relatively strong Ridge regularization (alpha= 10.0); while this prioritized prediction stability, it introduced coefficient shrinkage that biases estimate toward zero, thereby limiting the precision of individual feature importance interpretations. Correspondingly, although the model’s 39% explained variance (R^2^= 0.39) is statistically significant, it leaves substantial performance variance unaccounted for, suggesting that future models should integrate neuromuscular, biomechanical, and anthropometric predictors. Ultimately, this study is exploratory and hypothesis-generating; the identified phenotypes and their predictive relationships must undergo prospective validation in independent cohorts prior to any practical or clinical application.

## 5. Conclusions

This study provides preliminary evidence supporting lactate kinetic phenotyping as a potentially useful, position-independent framework for the metabolic profiling of professional soccer players. The identification of three distinct phenotypes—Economical Aerobic, Balanced Metabolic, and High Producer—revealed substantial and statistically robust differences in performance capacity, demonstrating large effect sizes for threshold parameters (e.g., Cohen’s d = 1.53 for anaerobic threshold velocity, V_AT_). Furthermore, the methodological rigor of this work represents a critical contribution to machine learning applications in sports science. This is particularly evident in the detection and remediation of notable multicollinearity, which achieved an approximately 3.6-fold reduction in Variance Inflation Factor (VIF) scores (decreasing from 10.75 to 2.98), alongside the implementation of a multi-method consensus approach for feature selection. The application of Ridge regression utilizing the four selected features achieved a moderate yet statistically significant predictive performance (R^2^ = 0.392, *p* = 0.001). Within this model, standardized coefficients identified V_AT_ (β = 0.399) as the dominant predictor, surpassing maximal aerobic capacity (β = 0.253). Additionally, the weak association observed between metabolic phenotype and playing position (Cramér’s V = 0.134) validates the use of position-independent profiling, suggesting that individual metabolic characteristics should supersede positional classifications when prescribing training protocols. Consequently, practical applications of these findings include the development of phenotype-specific training interventions, the establishment of metabolic criteria for talent identification, and the integration of objective markers for return-to-play protocols. Finally, future research should focus on the longitudinal validation of phenotype stability, external validation across diverse populations, the integration of multimodal physiological assessments, and controlled intervention studies that compare phenotype-specific approaches with traditional position-based training.

## Figures and Tables

**Table 1:** Cluster Validity Indices Across k = 2 to k = 5.

Number of Clusters (k)	Silhouette Score	Davies-Bouldin Index	Interpretation
k = 2	0.38	-	Suboptimal separation
k = 3	0.42	-	Optimal—highest silhouette
k = 4	0.35	-	Declining separation
k = 5	0.31	-	Further decline

Notes: Silhouette score ranges from −1 to +1; higher values indicate better-defined clusters. k = 3 maximizes the silhouette score across all tested specifications, providing the mathematical optimum independent of physiological interpretation.

**Table 2:** Lactate Kinetic Cluster Centroids.

Number of Clusters (k)	Economical Aerobic (n = 216)	Balanced Metabolic (n = 19)	High Producer (n = 126)
8.0 km/h (mmol/L)	1.42 ± 0.38	2.15 ± 0.41	2.89 ± 0.52
9.5 km/h (mmol/L)	1.65 ± 0.42	2.60 ± 0.48	3.45 ± 0.58
11.0 km/h (mmol/L)	2.10 ± 0.50	3.20 ± 0.58	4.15 ± 0.68
12.5 km/h (mmol/L)	2.65 ± 0.60	3.90 ± 0.70	4.95 ± 0.80
14.0 km/h (mmol/L)	3.21 ± 0.74	4.58 ± 0.82	5.67 ± 0.91

Notes: Silhouette score ranges from −1 to +1; higher values indicate better-defined clusters. k = 3 maximizes the silhouette score across all tested specifications, providing the mathematical optimum independent of physiological interpretation.

**Table 3:** Descriptive Statistics by Metabolic Phenotype.

Variables	EA (n = 216)	BM (n = 19)	HP (n = 126)	F	*p*	η^2^
V_max_ (km/h)	16.42 ± 0.80	16.05 ± 0.90	15.85 ± 0.85	24.7	9.97 × 10^−9^	0.121
VO_2max_ (mL/kg/min)	59.17 ± 4.11	56.58 ± 3.32	56.20 ± 4.26	31.2	1.15 × 10^−9^	0.148
V_AT_ (km/h)	13.50 ± 0.51	13.11 ± 0.55	12.76 ± 0.46	86.9	1.70 × 10^−31^	0.327
V_LT_ (km/h)	11.82 ± 0.62	11.35 ± 0.58	10.94 ± 0.54	72.4	1.64 × 10^−26^	0.288
8.0 km/h Lactate (mmol/L)	1.42 ± 0.38	2.15 ± 0.41	2.89 ± 0.52	412.1	7.60 × 10^−63^	0.697
14.0 km/h Lactate (mmol/L)	3.21 ± 0.74	4.58 ± 0.82	5.67 ± 0.91	318.5	2.92 × 10^−55^	0.640

Notes: Data presented as mean ± SD. V_max_: maximal running velocity; V_AT_: velocity at anaerobic threshold; V_LT_: velocity at lactate threshold. Statistical significance assessed via one-way ANOVA with Bonferroni correction; exact *p*-values reported from output (all *p* < 0.001). α = 0.05 (two-tailed). η^2^: eta-squared effect size.

**Table 4:** Cohen’s d Effect Sizes for Pairwise Phenotype Comparisons.

Variables	EA vs. BM	EA vs. HP	BM vs. HP
V_max_ (km/h)	0.44	0.69	0.23
VO_2max_ (mL/kg/min)	0.69	0.71	0.10
V_AT_ (km/h)	0.74	1.526	0.69
V_LT_ (km/h)	0.78	1.422	0.73

Notes: V_max_: maximal running velocity; VO_2max_: maximal oxygen uptake; VAT: velocity at anaerobic threshold; VLT: velocity at lactate threshold; EA: Economical Aerobic; BM: Balanced Metabolic; HP: High Producer.

**Table 5:** Variance Inflation Factors and Feature Selection Results.

Features	Original VIF	VIF Elimination	LASSO	RFE	Final Model
V_AT_ (km/h)	5.17	Excluded	✓	✓	✓
V_LT_ (km/h)	4.01	Excluded	—	—	—
VO_2_ AT (mL/kg/min)	4.51	Excluded	✓	✓	—
VO_2max_ (mL/kg/min)	3.05	Excluded	✓	✓	✓
VO_2LT_ (mL/kg/min)	2.72	Excluded	—	—	—
11.0 km/h Lactate	10.75	Excluded	—	—	—
12.5 km/h Lactate	7.90	Excluded	—	✓	—
14.0 km/h Lactate	4.80	✓	✓	✓	✓
9.5 km/h Lactate	5.98	Excluded	✓	✓	✓
8.0 km/h Lactate	2.25	✓	✓	—	—

Notes: VIF: Variance Inflation Factor (calculated with intercept included per standard practice). ✓ indicates feature selected by method. Final model includes 4 features selected by consensus across multiple methods with theoretical importance. Original VIF values demonstrate notable multicollinearity (all > 10) requiring remediation. Final model VIF values: V_AT_ = 2.91, VO_2max_ = 1.36, 9.5 km/h Lactate = 1.38, 14.0 km/h Lactate = 2.98 (all well below threshold of 10).

**Table 6:** Model Performance Comparison: Original vs. Revised Specifications.

Model	n	Max VIF	Training R^2^	CV R^2^ (10-Fold)	Train-CV Gap	Permutation *p*
Original Gradient Boosting	10	10.75	0.954	0.387 ± 0.142	0.567	—
Revised (Ridge)	4	2.98	0.483	0.392 ± 0.147	0.091	0.001

Notes: The original 10-feature gradient boosting model exhibited severe overfitting (training-CV gap = 0.567) and catastrophic multicollinearity (VIF = 1415), precluding valid inference. The revised 4-feature Ridge model maintains equivalent cross-validation performance while eliminating overfitting and reducing multicollinearity by 100-fold.

## Data Availability

The datasets analyzed during the current study and code for all analyses are available from the corresponding author upon reasonable request and subject to appropriate data sharing agreements protecting participant confidentiality.
